# Grasping the Agent’s Perspective: A Kinematics Investigation of Linguistic Perspective in Italian and German

**DOI:** 10.3389/fpsyg.2017.00042

**Published:** 2017-02-07

**Authors:** Claudia Gianelli, Michele Marzocchi, Anna M. Borghi

**Affiliations:** ^1^Division of Cognitive Sciences, University of PotsdamPotsdam, Germany; ^2^Department of Psychology, University of BolognaBologna, Italy; ^3^Institute of Cognitive Sciences and Technologies, National Research CouncilRome, Italy

**Keywords:** perspective taking, action, language comprehension, motor chains, motor system, motor resonance, pronouns, action verbs

## Abstract

Every day, we primarily experience actions as agents, by having a concrete perspective on our actions, their means and goals. This peculiar perspective is what allows us to successfully plan and execute our actions in a dense social environment. Nevertheless, in this environment actions are also perceived from an observer’s perspective. Adopting such a perspective helps us to understand and respond to other’s people actions and their outcomes. Importantly, similar experiences of being agent and observer occur also when actions are not physically acted/perceived but are merely linguistically shared. In this paper we present two exploratory studies, one in Italian and one in German, in which we applied a direct comparison of three singular perspectives in combination with different verb categories. First, second and third person pronouns were combined with action and interaction verbs, i.e., verbs implying an interaction with an object – e.g., grasp – or an interaction with an object and another person – e.g., give. By means of kinematics recording, we analyzed participants’ reaching-grasping responses to a mouse while they were presented with the different combinations of linguistic stimuli (pronouns and verb type). Results of Experiment 1 on reaching show that, when they are preceded by YOU, interaction verbs reached the velocity peak earlier than action verbs, since a further motor act will follow. Thus pronouns influence perspective taking and while comprehending language we are sensitive to the motor chain organization of verbs. The absence of the same effects in Experiment 2 is likely due to the fact that, being the pronoun in German mandatory, it is perceived as less salient than in Italian. Overall our result supports the idea that language is grounded in the motor system in a flexible way, and highlights the need for cross-linguistic studies in the field of embodied language processing.

## Introduction

Increasing evidence supports the notion that motor processes take place in our brains while we are either observing actions being made by others, or just hearing the verbal description of those actions. In particular, a large amount of data has recently shown that an activation of the motor system (a *motor resonance* process) is present very early during language comprehension, as revealed by physiological, neuro-imaging, and behavioral studies (for reviews see Fischer and Zwaan, 2008; Toni et al., 2008; Jirak et al., 2010; Glenberg and Gallese, 2012; Meteyard et al., 2012), as well as by computational work (for a review, see Borghi and Cangelosi, 2014). Different methodologies have contributed to characterize this motor activation, supporting the idea that it is not just a side effect of motor imagery but a constitutive part of language comprehension (for a review of the debate on this issue see Fischer and Zwaan, 2008; Mahon and Caramazza, 2008; Toni et al., 2008; Barsalou, 2016). Within behavioral studies, a special role has been played by studies in which kinematics of response movements were recorded (e.g., Boulenger et al., 2006; Dalla Volta et al., 2009; Scorolli et al., 2009; Borghi et al., 2010; Gianelli et al., 2013). By combining the presentation of simple action-related linguistic stimuli (e.g., single verbs, word pairs, short sentences) with a motor task (e.g., reaching-grasping or lifting objects) these studies showed early effects of language processing on motor planning and execution, implying the activation of sensorimotor representations corresponding to the semantic content of linguistic stimuli. However, and despite increasing evidence, the debate regarding the exact timing and nature of the activation of these language-induced sensorimotor representations is still open (see Papeo and Caramazza, 2014).

One open issue regards whether and how these representations are (1) flexible and (2) detailed in terms of the motor components they activate. The present study aims at providing some exploratory data regarding both flexibility of perspective and level of detail of the action components that different perspectives can generate with the help of the powerful tool of kinematics analysis. To this aim, we created a set of stimuli composed of sentence fragments combining a typical perspective-related device, i.e., pronouns, and two categories of action verbs.

Despite being a crucial process in our social interactions (Gallese, 2006; Keysers and Gazzola, 2006), perspective has so far not been a major target of embodied research in the language domain. In contrast, studies on action observation have underlined the importance of perspective, in particular of first-person perspective (Vogt et al., 2003; Jackson et al., 2006; Schütz-Bosbach et al., 2006; Bruzzo et al., 2008; Gianelli et al., 2008).

As pointed out by a recent review (Beveridge and Pickering, 2013), most embodied language studies seem to implicitly assume that readers and listeners activate a first-person – that is an agent – perspective during language comprehension. Likely, this is due to the fact that evidence on motor activation is mainly collected using isolated verbs, rather than verbs embedded within sentences and discourses. But since this assumption is not explicit, the possibility that other perspectives might be activated has not been thoroughly investigated and existing evidence is unclear. Probably also due to the implicit focus on the agent’s perspective, pronouns have not been extensively investigated in the recent studies focusing on the motor grounding of language, with the exception of some linguistic studies (MacWhinney, 2005). Pronouns, however, are important as they have at least a double role: first, they allow us to understand who is performing an action (the agent); second, they give us information regarding the involvement of someone/something else (e.g., patient object) in the action. To our knowledge only a few studies contrasted the motor effects of first and third person action verbs, but they obtained contrasting results. For example, Tomasino et al. (2007) found no difference between first and third person German action verbs with an fMRI study, while in a more recent TMS study Papeo et al. (2011) found a modulation of motor-evoked potentials during processing of first-person but not of third-person Italian action verbs. Furthermore, these results were difficult to compare due to the different languages (German, Italian), to the different techniques (fMRI, TMS) and to the different task. Other TMS studies, such as the study by Buccino et al. (2005) and a subsequent controlled replication of it (Gianelli and Dalla Volta, 2015) found that stimulation of the hand motor cortex while listening to third-person Italian action verbs do indeed modulate motor-evoked potentials as compared to verbs involving other effectors and abstract verbs. However, the latter studies used complete sentences with third-person pronouns instead of infinitive verbs (or sentence fragments) but without direct perspective manipulation. In addition, they used a passive listening task of sentences with only implicit pronoun and the stimulation of the motor cortex occurred when perspective was not yet fully elicited (e.g., in sentences like “cuciva la gonna/she sewed the skirt”). For this reason, although providing evidence in conflict with Papeo et al. (2011), these studies cannot provide final conclusions on this issue.

A small number of behavioral studies have addressed a similar topic but with tasks that did not directly involve the motor system. Brunyé et al. (2009) used a picture-verification task to investigate the perspective adopted when reading action sentences. They compared perspectives implied by the different pronouns (I, You, He) and showed that participants automatically activate an internal perspective when directly addressed as agents (You), whilst activating an observer perspective in the case of He and I pronouns. Interestingly, the same results were obtained also when the task did not explicitly involve a mental simulation, for instance with a memory task (Ditman et al., 2010). However, in this study the linguistic perspective was directly matched with a visual perspective and the authors did not use a motor task.

Differently from passive TMS and fMRI studies, and from behavioral ones, investigations using an explicit motor task might be more informative, as they clearly pose a strong focus on the agent’s perspective by requesting participants to perform simple movements as response. However, evidence under this respect is still very limited. For instance, Gianelli et al. (2011) used a novel version of the Action-sentence Compatibility Effect (ACE, Glenberg and Kaschak, 2002) showing that shifting perspective from first to third person was sufficient to prevent the activation of sensorimotor representations, abolishing the behavioral ACE. Critically, the ACE was restored by adding a virtual “body” that allowed participants to know “where” to put themselves in space when taking the third person perspective, thus demonstrating that motor embodied processes are space-dependent. In addition, Gianelli et al. (2013) recently showed how the social and spatial perspective conveyed by the physical presence of another participant and by linguistic productions, affect a simple reaching-grasping task. However, the focus of these studies was either on complete sentence comprehension (Gianelli et al., 2011) or employed a complex manipulation of social intentions (Gianelli et al., 2013). Moreover, in both studies the agent’s perspective (i.e., the actual motor information) was not manipulated and the interplay between linguistic and motor perspective was only limited.

The present study addressed the role of perspective by using sentence fragments starting with the three singular personal pronouns, You, He/She and I.

The first manipulation we introduced, i.e., the use of three pronouns, allowed us to disentangle two alternative hypotheses. In the first one, language structure would exactly reflect the action structure regardless of linguistic perspective, as assumed by standard embodied cognition theories. If this is the case, then while reading simple pronoun-verb pairs we should automatically activate an agent-independent sensorimotor representation. This would imply that similar motor effects should be detected regardless of the pronouns and thus linguistic perspective. In the other, a more flexible view of embodied cognition would predict the activation of different motor patterns as implied by different linguistic pronouns and hence perspectives. If this is the case, then the pronoun YOU would likely activate the agent’s perspective thus modulating motor responses, according to the motor information given by the motion verbs (i.e., action vs. interaction pattern). On the contrary, the pronoun I should be perceived as conveying an observer’s perspective, thus activating motor information at a lesser and/or different extent since no contextual information was given. Similarly, the HE/SHE pronoun should activate a completely external perspective, thus producing no modulation of kinematics parameters at all.

The second manipulation we introduced concerns the kinds of action verbs we selected. We addressed the possibility to detect if the agent’s perspective is activated, and how detailed it is, by manipulating the motor nature of the action verbs composing our sentence fragments. In particular, we decided to focus on the hypothesis that actions are structured into chains of motor acts, informed by the overall action goal. A variety of results obtained initially with monkeys and then with humans (Fogassi et al., 2005; Iacoboni et al., 2005; Cattaneo et al., 2007; Boria et al., 2009; Fabbri-Destro et al., 2009) show that a mechanism of motor chains constitutes one of the basic structures of the motor system. A chain of motor acts is informed by the final action goal, thus motor acts are organized in the chain so that each of them depends on the successive and all depend on the last. Goals characterize both single motor acts and actions as a whole (for a computational model of chained organization in language, see Chersi et al., 2010). Because of these basic properties, the motor chain structure is an ideal target for disentangling whether and how an agent perspective is activated during linguistic processing of actions.

To this aim, we constructed very simple sentence fragments composed by a pronoun and a motion verb, with verbs being divided into two main categories, that we called *action verbs* and *interaction verbs* (AVs, IVs) (e.g., grasp vs. give). Action and interaction verbs differed according to various dimensions (for a similar approach, see Kemmerer and Gonzalez-Castillo, 2010). First, the two categories differed for the relations they describe and involve: in one case the direct relation subject-object, in the other case the triadic relation subject-object-other subject. Second, they differed for how these relations imply different goals: AVs are actions which may stand alone and whose final goal might be the sole manipulation of an object, whilst IVs directly imply the interaction with another person. Third, they differed as to the organization in motor chains: AVs and IVs share the motor act of reaching-grasping an object, while they differ for the last act of the sequence, the one determining all the others, which might imply or not the presence of another person. Thus, even if the first part of the motor chain is common, the chain is embedded within two different goals, one of which involves the interaction with another person. Previous kinematics literature has shown higher accuracy with actions guided by a social intention (Becchio et al., 2008; Ferri et al., 2011; Gianelli et al., 2013; Scorolli et al., 2014). However, to our knowledge the “social accuracy” effect has been never investigated distinguishing in the linguistic domain. We predict that IVs lead to higher accuracy compared to AVs in correspondence with the planning of the final motor act, the one that implies an interaction with another person and that qualifies the overall goal of the fragment.

In the experiment participants were required to reach and grasp an object (the mouse) while reading a sentence fragment composed by a pronoun and a verb. The task we chose was designed in order to induce participants to pay attention to both the pronoun and the verb: for this reason, once identified the verb and grasped the mouse, they were required to continue the movement and to click the mouse if the pronoun and the verb matched (“io prendevo,” I took) and to refrain from continuing the movement if the pronoun and the verb did not match (“io prendeva,” You took, wrong in Italian since the pronoun refers to the first person and the verb to the third one). The task allowed us to investigate the development of the effect of linguistic stimuli on the overt action of reaching and grasping, through the analysis of its fine-grained kinematics aspects. Our general aim was to disentangle the final effects of the two components, pronouns and verbs, and at the same time to understand how their effects are combined producing a modulation of various phases of movement kinematics.

First, we intend to test if the pronouns affect the adopted perspective, influencing the motor response. If the agent’s perspective is automatically activated, regardless of linguistic perspective, then motor effects should be present in all conditions and thus not significantly differ among these. If the activation of sensorimotor representations is instead flexible, we should then find effects only with pronouns that activate a first-person, that is the agent’s, perspective (i.e., YOU).

Second, we intend to test whether the perspective activated by pronouns is modulated by the motor chains implied by the two kinds of verbs, influencing the motor responses. If the perspective-related sensorimotor activations are general and abstract, then no effect of verb category should be detected. If, on the contrary, the degree of activation is such that the typical motor chain organization is activated, then processing AV or IV verbs should produce detectable motor outputs. In particular, the different structure of AV and IV should be mapped onto specific parameters of the motor response, i.e., those connected with the velocity peak and its latency since they are typically affected by increased accuracy requirements (i.e., namely with those actions that IVs describe).

## Experiment 1

### Methods

#### Participants

Twelve women, aged 18–28, participated in this study, and were recruited among Communication students at the University of Bologna. All participants were right-handed by self-report, native Italian speakers and reported normal or corrected-to-normal vision. All were naive as to the purpose of the experiment and gave their informed consent to the experiment, which was approved by the local Ethics Committee of the University of Bologna.

#### Procedure

The experiment took place in a soundproof room. Participants sat in front of a laptop, whose LCD monitor was set on a temporal resolution of 60 Hz. The distance between hand and monitor was of 60 cm. Participants started placing their right hand on the table in a pinch position. The target of the subsequent reaching-grasping movement was a mouse, placed in line with the hand of the participant, at a distance of 33 cm. The final position for the mouse movement was set at 50 cm. The hand movement was performed on the right of the laptop, at a distance of 5 cm. This allowed participants to easily perform the movement and look at the screen.

#### Stimuli

Stimuli consisted of ten Italian verbs referring to manual actions (see **Table 1**). We selected five proper “action” verbs (AVs), which involved a direct relation subject-object (e.g., *to grasp)* and five “interaction” verbs (IVs), involving at least a relation subject-object-subject (e.g., *to give*). A sample of sixteen students evaluated these verbs on two 7-point scales, one aimed to rate how much the verbs implied a relation subject-object (action scale), the other how much the verbs involved another person (interaction scale). An ANOVA performed on the mean ratings (considering two types of verbs and two type of ratings) showed a significant interaction [*F*(1,15) = 15, 2, *MSE =*21.39, *p*= 0.001) between verb type and rating. As predicted, AVs obtained higher values in the action scale, whilst IVs obtained higher values in the interaction scale. Two additional independent groups of ten students each evaluated the same verbs on two 7-point scales for concreteness and abstractness. An ANOVA performed on the mean ratings (considering two types of verbs and two scales) showed a main effect of scale: in general all verbs were evaluated as more concrete than abstract [*F*(1,9) = 22.296, *MSE =*14.16, *p* < 0.002], an expected result since we focused on choosing verbs with a specific action-relatedness. More interestingly, a significant interaction of verb type and scale was also detected [*F*(1,9) = 25.857, *MSE =*29.93, *p* < 0.001]. While the evaluation of IVs tended to be constant along the two scales, (*M* = 4.36 vs. *M* = 3.82), AVs were evaluated higher in the concreteness scale (*M =*5.8 vs. *M* = 2.88). However, a Newman–Keuls *post hoc* test revealed that AVs and IVs did not significantly differ in the abstractness scale (*p*> 0.05), but they differed in the concreteness scale (*p*< 0.05). This was expected, since we selected AVs as specifically related to object interaction and manipulation, whereas IVs imply a relation with another subject, which can be considered as less concrete. Furthermore, IVs are often related to abstract sentences or expressions, which could explain a tendency to associate them with more abstract contexts. Nevertheless, both AVs and IVs had low scores in the abstractness scale and did not significantly differ: it seems then unlikely that the observed effects were due to this property and not to the experimental manipulations.

**Table 1:** Complete list of stimuli in Experiments 1 and 2.

Infinitive	Verb	1st	2nd	3rd	English
**Experiment 1-Italian**					
afferrare	AV	io afferravo	tu afferravi	egli afferrava	to grasp
alzare	AV	io alzavo	tu alzavi	egli alzava	to raise
portare	AV	io portavo	tu portavi	egli portava	to carry
prendere	AV	io prendevo	tu prendevi	egli prendeva	to take
sol levare	AV	io sollevo	tu sollevavi	egli sollevava	to lift up
consegnare	IV	io consegnavo	tu consegnavi	egli consegnava	to deliver
dare	IV	io davo	tu davi	egli dava	to give
offrire	IV	io offrivo	tu offrivi	egli offriva	to offer
porgere	IV	io porgevo	tu porgevi	egli porgeva	to hand
scambiare	IV	io scambiavo	tu scambiavi	egli scambiava	to exchange
**Experiment 2-German**					
packen	AV	ich packe	du packst	er packt	to pack
g reif en	AV	ich greife	du g re ifst	er greift	to grasp
heben	AV	ich hebe	du hebst	er hebt	to lift
holen	AV	ich hole	du hoist	er holt	to get
ergreifen	AV	ich ergreife	du erg re ifst	er ergreift	to seize
schnappen	AV	ich schnappe	du schnappst	er schnappt	to grab
fassen	AV	ich fasse	du fasst	er fasst	to take
bringen	IV	ich bringe	du bringst	er bringt	to bring
reichen	IV	ich reiche	du reichst	er reicht	to hand
liefern	IV	ich liefere	du lieferst	er liefert	to supply
tauschen	IV	ich tausche	du tauschst	er tauscht	to exchange
geben	IV	ich gebe	du gibst	er gibt	to give
bieten	IV	ich biete	du bietest	er bietet	to offer
stiften	IV	ich stifte	du stiftest	er stiftet	to donate

For each verb we identified the isolation point (IP), intended as the minimum part of the verb required to understand it and to differentiate it from similar verbs. In our stimuli the IP corresponded to the verbal stem, as showed in **Table 1**. The final set of stimuli was fully balanced for syllables, length, IP duration, and written lexical frequency (ColFIS, Bertinetto et al., 2005).

Each verb was presented in written form in the three singular persons of the Italian past tense in order to compose sentence fragments. In Italian the pronoun can be omitted, as the verb contains information on the person. However, in our case, using both the pronoun and the past tense, we obtained a double reference to the agent. The final set of stimuli comprised 10 verbs, each presented once in combination with one of the three pronouns (30 critical trials). We inserted also 10 catch-trials, i.e., verbs in the same tense as the others but incorrect for the correspondence verb-subject, e.g., “io portava”: in this case the explicit subject is a first person pronoun while the verb refers to the third person. Catch-trials required participants to refrain from completing the movement and were not analyzed further. The experiment was run in a single block of 40 trials.

#### Experimental Design

**FIGURE 1:**
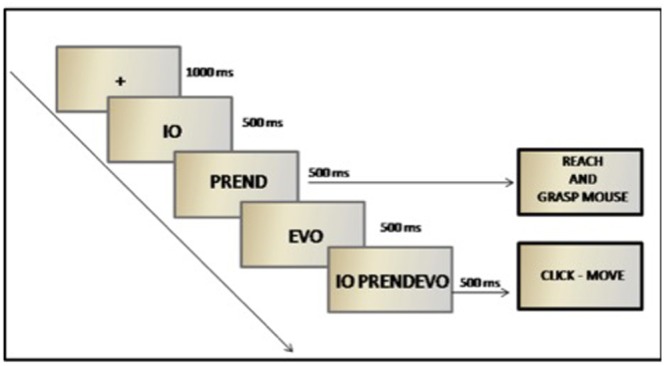
**Experimental procedure**.

As described in **Figure 1**, each trial started with a fixation cross (1000 ms). Then a pronoun was shown for 500 ms, followed by the first part of the verb (e.g., “prend”) displayed for 500 ms. Subjects were required to pay attention to both the pronoun and the verb, and once they recognized the verb they had to start moving as fast as possible to reach for and grasp the mouse in front of them. During the movement the verb was completed with its suffix (e.g., “evo”) (500 ms). This time was sufficient to accomplish the movement at about the same time in which the complete stimulus “io prendevo” (I took) was presented (time limit of 500 ms). Participants held the hand on the mouse till they decided whether the sentence was correct or not. If correct, they had to click on the left button and then move the mouse to the final position. Otherwise, they had to refrain from moving.

#### Data Recording and Kinematic Analysis

Movements of the participant’s right hand were recorded using the 3D-optoelectronic SMART system (BTS Bioengineering, Milano, Italy), by means of three infrared cameras at a sampling rate of 60 Hz. Recorded data were filtered using a linear smoothing low pass filter and stored for oﬄine analysis. We used three markers, one applied on the wrist, and the other two on the nail of the index and of the thumb finger respectively.

We considered two components of movement, reaching and grasping, and for each of them we identified different parameters. We avoided considering the act of giving/placing of the mouse due to the high variability of the performed movements.

For the reaching component we analyzed the behavior of the marker placed on the wrist. We considered the reach time, the time to velocity peak (latency), the % of time to velocity peak (normalized with respect to the reach time), and the amplitude of the velocity peak.

To analyze the grasp component we considered the time course of the distance between the two markers posed on the index and on the thumb finger. We analyzed the following parameters: grasp time, maximal finger aperture, time to maximal finger aperture (latency) and percentage of time to maximal finger aperture by means of the software Smart Analyzer and a customized Matlab script. We followed rules and conventions defined by Gianelli et al. (2008) to analyze the different components; in summary: based on the spatial resolution of the system, the reach beginning was defined as the first frame in which the displacement of the wrist marker exceeded 0.3 mm in all Cartesian axes; conversely, to determine the reach end, we first defined the first frame after velocity peak in which the displacement of the reach marker was <0.3 mm along the three axes. The frame (x, y, or z) closer to the grasp end time was selected as reach end. As to the grasp, grasp beginning was defined as the first frame in which the distance between the two markers exceeded 0.3 mm, while grasp end corresponded to the first frame after maximal finger aperture in which the distance between the two markers was less than 0.3 mm. Since reach time and grasp time were defined separately for the two component, normalization with respect to these measures was performed separately for reach and grasp parameters (a similar normalization procedure was applied for instance in Gentilucci, 2002; Gianelli et al., 2008; Ferri et al., 2011).

#### Data Analysis

Trials with errors (e.g., in the linguistic task, moving when not requested or refraining to do it, anticipated movements, impossibility to correctly separate the reaching of the mouse and the placing during data analysis, etc.) were marked during kinematics analysis and rejected. Participants showing less than 50% of valid trials were excluded from the statistical analysis.

Data analysis was performed only on critical trials (i.e., catch-trial were not analyzed). A repeated measures ANOVA was conducted on the mean values of participants’ reaching parameters, considering Verb (AV vs. IV) and Pronoun (I, YOU, HE) as within-subjects factors. For each significant parameter we report also an estimate of the effect size (η_p_^2^).

### Results

The percentage of errors was negligible (under 1.5%), thus participants correctly understood the word pairs in order to perform the grammatical task and correctly performed the requested motor response. No participant was excluded from data analysis. All results are summarized in **Table 2**.

**Table 2:** Summary of results in Experiment 1 and 2, all times are expressed in ms.

	l_Action	You_Action	He_Interaction	I_Interaction	You_Interaction	He_Interaction
**Experiment 1 – Italian**						
Reach time	742,6	685,6	744,8	705,1	729,8	720,7
Time to velocity peak	367,3	355,6	358,6	360,8	345,6	346,8
% Time to velocity peak	49,4	52,2	48,5	51,8	47,4	49,3
**Experiment 2 – German**						
Reach time	883,4	869,3	868,5	861,2	879,8	894,5
Time to velocity peak	280,0	277,0	283,0	272,3	276,1	281,0
% Time to velocity peak	31,6	31,7	32,3	31,6	31,0	31,2

#### Reaching Component

During the act of reaching we observed no significant main effects of Verb or Pronoun. However, the analysis showed a significant interaction Verb–Pronoun in the normalized % of time to velocity peak, *F*(2,22) = 6.48, *p*= 0.006, η_p_^2^ = 0.4. Following our predictions, *t*-test comparisons were then used to detect the differences between the two kinds of verbs (action vs. interaction) in combination with the three pronouns. A Bonferroni correction for multiple comparisons was applied, with a *p-*value fixed at 0.01. The result showed that the only significant difference was between AVs and IVs when preceded by the pronoun YOU, *t*(11) = 2.81, *p*= 0.008 (equivalent to a 4.8% difference between conditions). In this sense, IVs showed a shorter time to reaching the velocity peak as compared to AVs. This specific pattern is typically detected at the planning stage when a higher accuracy and the programming of a further motor act are required, as it was the case for IVs and not for AVs. This parameter is thus connected to the activation of the agent’s perspective, as activated in a conversational framework by the pronoun YOU. The pronoun I slightly modulated the motor responses but did not reach significance, *t*(11) = 1.84, *p* = 0.05. The same was true for the pronoun HE, as it did not modulate the motor responses at all, *t*(11) = 0.56, *p*= 0.3. No other parameters reached significance^1^.

**FIGURE 2:**
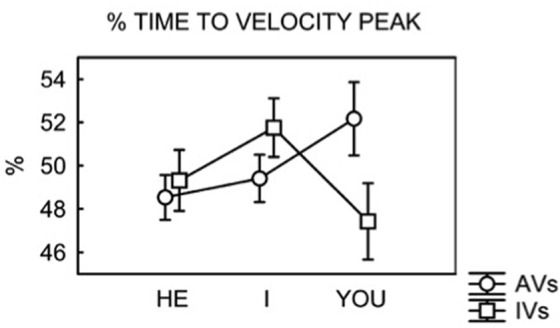
**% of Time to velocity peak, interaction between perspective and verb type**. Bars are SE.

The results indicate a specific contribution of the pronoun YOU in activating the agent’s perspective and thus modulating one key parameter in the reaching component (the normalized latency of velocity peak), whereas the I perspective did not show significant modulations (**Figure 2**).

#### Grasping Component

Repeated measures ANOVAs on grasping parameters showed that no parameter reached significance. In particular, the ANOVA on the time to maximal finger aperture (i.e., time between finger opening and the maximal aperture before grasping the object), did not reach significance, *F*(2,22) = 2.08, *p* = 0.148, η_p_^2^ = 0.2. At the qualitative level we can see that the YOU pronoun is the one which mostly modulates the differences between the two verbs, showing overall longer times for the pronoun YOU (*M* = 491 ms) than for the pronoun I (*M* = 441 ms).

### Discussion

The results of the study indicate that motor responses are influenced both by the perspective induced by the pronouns and by the different kinds of verbs. We namely found an interaction between the kind of pronoun and the kind of verbs in the analyses on the reaching component. When they were preceded by the YOU pronoun, Interaction verbs reached the velocity peak earlier than Action verbs; this determines a longer deceleration phase. The longer deceleration phase is compatible with the fact that the current motor act is influenced by the next one, i.e., that the action of grasping is influenced by the subsequent action of giving.

The difference we found between Interaction and Action verbs, when preceded by YOU, suggests that when adopting the agent perspective (recruited by the YOU pronoun) we are sensitive to the motor chain structure of verbs. Indeed, while with Action verbs the action terminates once the object is grasped, with Interaction verbs a further motor act follows, since the object has to be given to somebody else.

Furthermore, the finding that the YOU pronoun modulated reaching suggests that, once we read action verbs, we do not automatically assume the agent perspective, but that the adopted perspective is flexible and depends on the presented pronoun.

A research question remains, however, open. While our results demonstrate that in Italian the linguistically presented pronoun influences the motor system, it remains to be determined whether such an influence varies depending on the spoken languages. It is indeed possible that such an influence is present only in languages as Italian where the pronoun assumes salience when mentioned, since it is not mandatory. For this reason in Experiment 2 we adapted the same design and rationale to German stimuli. In the introduction of Experiment 2 we will explain in more detail why we choose to perform a study with a similar inspiration in German.

## Experiment 2

The results of Experiment 1 showed that the combination of pronouns and verbs affects movement execution in a way supporting the hypothesis that an agent’s perspective is flexibly activated only under certain conditions and not others (e.g., external perspective). In Experiment 2 we intended to produce a conceptual replication with the same design and rationale but with German stimuli and participants. The reason why we decided to compare Italian and German language is that the role played by the pronouns in the two languages is profoundly different. Italian is a language in which the explicit pronoun can be omitted as the verb already conveys this information (a pro-drop language); however, the relative position of the pronoun and the verb in the sentence is very strict. In German, instead, the use of the pronoun is mandatory, and is very often decisive for revealing the exact subject of a verb. Nevertheless, German speakers are used to a much more flexible sentence construction and word order: previous research, for instance, has shown how this flexibility makes easier for German speakers’ to comprehend and produce constructions, such as passive sentences, that result harder to process for other languages’ native speakers (see Armon-Lotem et al., 2016).

Both these characteristics can render the pronoun, when mentioned, less salient in German compared to Italian. Consistently with this interpretation, while previous data in Italian (our own, but also Papeo et al., 2011) seem to point to different motor activations according to different perspectives, the only data available in German (Tomasino et al., 2007) suggest that no difference is present between first and third person action verbs. Experiment 2 is therefore aimed ad investigating whether the same effects of language-induced perspective and on the motor responses we have found in Experiment 1 are present also in German, a language in which the pronoun is mandatory.

It is worth of notice that, even if we built the experiment in Italian and German starting from the same hypotheses and inspiration, the two experiments are not directly comparable. The choice to use Italian and German had indeed a consequence on the experimental stimuli we selected: in order to be able to correctly identify the verb IP, intended as the minimum part of the verb required to understand it, we had to choose verbs of different tenses in the two languages –past in Italian and present in German (see the method section of Experiments 1 and 2 for further information). Even if the two experiments are not directly comparable, Experiment 2 can be informative as to how participants process simple sentence fragments and the perspective information they convey, by producing a set of data obtained starting from the same hypotheses and inspirations in a language with different structural characteristics, such as German.

### Methods

#### Participants

In order to estimate the required sample size we used the effect size derived from Experiment 1 in order to establish the aimed sample size for Experiment 2. To this aim we used the software G^∗^Power (Version 3.1.6, University of Duesseldorf) procedure for repeated measures ANOVA and used the effect size estimated derived by the significant Verb^∗^Pronoun interaction in Experiment 1 (setting alpha at 0.05 and the desired power to 0.95). The resulted sample size of sixteen participants was thus used as a stopping rule for data collection in this experiment, with no replacement except in case of technical issues occurred during data recording (e.g., the participant is immediately rejected during the experiment because of the lack of a complete data set). In this case, we tested a total of nineteen participants, of which three did not provide a complete dataset because of technical issues – a sample of sixteen complete data sets thus entered data analysis. All participants were Psychology students at Potsdam University, native German speakers (all women, age 19–35), right-handed by self-report. As confirmed by a standard Edinburgh questionnaire (Oldfield, 1971), they had normal or corrected-to-normal vision and gave their written informed consent as requested by the local Ethics procedures. They took part to the experiment in exchange of course credits.

#### Procedure

The experiment took place in a soundproof room. Participants sat in front of a PC with the monitor set to a temporal resolution of 60 Hz. The distance between hand and monitor was of 60 cm. Participants started placing their right hand on the table in a pinch position. The target of the subsequent reaching-grasping movement was a mouse, placed in line with the hand of the participant, at a distance of 35 cm. The final position for the mouse movement was set at 50 cm.

#### Stimuli

Experiment 2 was built on the same principles and categories of experiment one. First, verbs pertaining the two categories were selected resulting in seven AVs and seven IVs (see **Table 1**). In order to select the stimuli, a sample of 34 psychology students recruited at the University of Potsdam filled an online questionnaire in exchange of course credits. All participants were native German speakers and were asked to evaluate each verb (given in the infinitive form) according to the same 7-point scales used for Experiment 1: action and interaction scales, as well as concreteness and abstractness ones.

As in Experiment 1, we first compared the results of the action vs. interaction scales by means of a 2^∗^2 ANOVA with verb type (action, interaction) and rating scale (action, interaction) as factors. The results showed a significant main effect of verb type [*F*(1,33) = 56, 22, *MSE =*13.03, *p* < 0.001] and an interaction between verb type and rating scale, [*F*(1,33) = 47, 52, *MSE =*18.46, *p* < 0.001]. While both verbs were similarly rated along the action scale, IVs were rated significantly higher in the interaction scales as compared to AVs [paired-sample *t*-test comparison, *t*(33) = -588, *p* < 0.001].

In a second ANOVA we compared the results of the concreteness vs. the abstractness scales by means of 2^∗^2 ANOVA with verb type (action, interaction) and rating scale (concrete, abstract) as factors. As in the first experiment, a main effect of scale [*F*(1,33) = 195.203, *MSE =*114.706, *p* < 0.001] shows that overall verbs were evaluated higher in the concreteness than in the abstractness scale, as we selected verbs with a specific action-relatedness. This main effect seems to drive the significant interaction we also found between verb type and rating scale [*F*(1,33) = 8.901, *MSE =*0.972, *p* = 0.005]. As in experiment one, AVs and IVs did not differ along the abstractness scale [*t*(33) = -1.527, *p* = 0.136], while they differed along the concreteness scale with AVs being evaluated slightly higher in the concreteness scale [*M* = 4.279 vs. 4.074, *t*(33) = 3.956, *p* < 0.001]. The same considerations regarding this scale for Experiment 1, hold for these stimuli as well.

No verb was excluded at this stage and all fourteen verbs entered one last linguistic evaluation with the aim of being matched for syllables, length, and written frequency (database: dlexDB). As in the case of experiment one, we selected a tense in which it would be acceptable to split the verb in two between the stem of the verb and the suffix that contains the information relative to tense and subject. To this aim, we selected the present tense of regular German verbs, as it fulfills our requirements, e.g., “Ich greife” vs. “Du greifst” vs. Er greift” (I grasp, You grasp, He grasps). As a clarification, past tense would not have worked, being respectively “Ich griff,” “Du griffst,” “Er griff,” and for different reasons the same holds for composite forms as the perfect. As we already noticed, compared to Experiment 1, in German the presence of both the personal pronoun and the subject information given by the verb is mandatory (all verbs are listed in **Table 1**).

Each verb was presented in written form in the three singular persons of the German present tense in order to compose sentence fragments. The final set of stimuli comprised 14 verbs, each presented twice in combination with one of the three pronouns (84 critical trials). We inserted also 16 catch-trials, i.e., verbs in the same tense as the others but incorrect for the correspondence verb-subject, e.g., “er greifst”: in this case the explicit subject is a third person pronoun while the verb refers to the second person. Catch-trials required participants to refrain from completing the movement and were not analyzed further. The experiment was run in a single block of 100 trials.

#### Experimental Design

The procedure was the same as described for Experiment 1: each trial started with a fixation cross (1000 ms). Then a pronoun was shown for 500 ms (“Ich”) followed by the first part of the verb (“greif”) displayed for 500 ms. Subjects were required to pay attention to both the pronoun and the verb, and once they recognized the verb they had to start moving as fast as possible to reach for and grasp the mouse in front of them. During the movement the verb was completed with its suffix (“e”) (500 ms). This time was sufficient to accomplish the movement at about the same time in which the complete stimulus “Ich greife” was presented (time limit of 500 ms). Participants held the hand on the mouse till they decided whether the sentence was correct or not. If correct, they had to click on the left button and then move the mouse to the final position. Otherwise, they had to refrain from moving.

#### Data Recording and Kinematic Analysis

Movements of the participant’s right hand were recorded by means of a 3D guidance tracking system (Trakstar, Ascension) with a sampling rate of 200 Hz, filtered using a linear smoothing low pass filter and then stored for oﬄine analysis.

The choice of the movement components and movement parameters were guided by the results of the first experiment. As Experiment 1 showed effects pertaining only the reach component of movement, we focused on the analysis of one sensor placed on the participants’ right wrist and analyzed parameters related only to this component. As in the first experiment, we analyzed the reach time, time to velocity peak (latency), % of time to velocity peak (normalized with respect to the reach time), and the amplitude of the velocity peak by means of a customized Matlab script. In this case, reach beginning and end were determined as the first and last frame in which the velocity was >1 mm/s. Normalization procedures were the same as in Experiment 1.

#### Data Analysis

Data analysis was the same as in Experiment 1 and it was performed only on the critical trials. Trials with errors (e.g., in the linguistic task, hence moving when not requested or refraining to do it, anticipated movements, impossibility to correctly separate the reaching of the mouse and the placing during data analysis etc.) were marked during kinematics analysis and rejected. Participants showing less than 50% of valid trials were excluded by statistical analysis. Statistical analysis was the same as in Experiment 1.

### Results

Three participants were excluded from statistical analysis based on the number of valid trials. The remaining thirteen participants entered the statistical analysis with a total of 92% of analyzed trials equally distributed across all participants and conditions (13 trials on average per condition).

#### Reaching Component

Statistical analysis (ANOVA) showed no significant main effect or interaction for any of the selected parameters (all *p*_s_ > 0.05). In particular, the critical parameter of % of velocity peak (significant in Experiment 1) resulted in a *F*(2,24) = 0.341, *p* > 0.7, η_p_^2^ = 0.028, with a difference as small as 0.6% between the you-action and you-interaction conditions (all data are summarized in **Table 2**). According to significance testing, then, no effect of perspective was detected in the second experiment, hence providing no evidence for a similar effect in the Italian and German experiments.

### Discussion

Experiment 2 aimed to verify whether the different pronouns and the two different kinds of verbs had an influence on motor response in German, a language chosen because, differently from Italian, pronouns are mandatory while the sentence construction is flexible. The results of Experiment 1 were not replicated. We will discuss the possible reasons of the missing effects in the Section “General Discussion.” To have a better idea of what happened in the two experiments, we analyzed them also using a Bayesian approach.

#### Exploratory Bayesian Analyses

As already shown, planned analyses for both experiments were based on classical null hypothesis significance testing (NHST) and relative estimation of effect size. Under this respect, Experiment 1 clearly showed a significant modulation of reaching parameters while Experiment 2 showed no significant effects. The significance tests thus leave the contribution of Experiment 2 unclear: how strong is the observed evidence against a modulation of kinematics parameters in German? Nevertheless, is the significant modulation observed in Experiment 1 substantial or just anecdotal?

In order to investigate these issues and complement our results, we performed an additional, exploratory analysis taking a Bayesian approach, with the aim to quantify the observed evidence in terms of odds ratio between the null and the alternative hypothesis. To this aim we report the results of two JZS Bayes factor ANOVA (using JASP, Rouder et al., 2012; Morey and Rouder, 2013; Love et al., 2015) with default prior scales, based on the data on the crucial parameter of % velocity peak for both experiments separately. In addition, and since the % of velocity peak is a normalized measure determined by the latency of velocity peak and the reach time, we tested these two parameters as well, although they did not show any difference in the significance tests.

For Experiment 1, the % of time to velocity peak shows a BF_10_ = 7.301 (that is a BF_01_ = 0.137) for the model comprising the interaction between the two factors, verb and subject type (as compared to the null) providing substantial evidence in support of the alternative hypothesis. In other terms, the observed data are seven times more likely to occur under H_1_. For Experiment 2, the same parameter produced a BF_10_ *=*0.23 for the same comparison (that is a BF_01_ = 4.378), providing no evidence in favor of the alternative hypothesis, with the observed data being far more likely to occur under H_0._ As to the other parameters, reach time showed comparable BFs in the two experiments (BF_10_ = 0.735 vs. 1.083, that is BF_01_ = 1.36 vs. 0.92), with the observed data almost equally likely to occur under H_0_ or H_1_. On the other side, the latency of velocity peak in Experiment 1 produced a BF_10_ = 0.420 (BF_01_ = 2.381) and in Experiment 2 BF_10_ = 0.203 (BF_01_ = 4,94), that is the observed data are more likely to occur two times and almost five times under H_0_ than H_1_ in both experiments.

## General Discussion

The results we found in the experiment in Italian and in German are quite different. We will first discuss the overall issue of whether language influences the motor system considering the results of the two studies. Then we will discuss more specific issues, i.e., the role of pronouns and verbs in light of the results of the first experiment. Finally we will outline the possible reasons why we found different results in the two languages.

### Language and Flexible Involvement of the Motor System

The results of our exploratory kinematics analysis in Experiment 1 showed the presence of distinct motor patterns as influenced both by the perspective elicited by the pronouns and the motor chains elicited by action verbs.

The interaction between verbs and pronouns found in Experiment 1 suggests that the effect of modulation due to language occurs early during the actual movement and is evident in a range of 300–350 ms after stimuli presentation. Interestingly, the parameter in which we found a modulation is connected to the velocity peak. We know well that the velocity peak is the main parameter which is defined in movement planning and it is susceptible to be affected by the various factors (motor factors as in Gentilucci et al., 1997; Dalla Volta et al., 2009 or social factors as in Ferri et al., 2010) under which the movement is executed. Consequently, the effect of our stimuli on this crucial parameter suggests that our stimuli mainly affected the planning stage of action. This early influence of linguistic processing on the motor system suggests that the activation of the motor system is not due to late-occurring imagery processes; it is therefore consistent with the view according to which the activation of the motor and sensorimotor cortices is not just a side effect but effectively contributes to language comprehension.

While the interaction Verb–Pronoun found in Experiment 1 clearly indicate that pronouns and verbs differently influence the motor system, the absence of a perspective-related modulation following German stimuli might point to the activation of agent-independent sensorimotor representations. However, we do not believe that we can draw such a conclusion. Indeed, the absence of a baseline/reference condition (e.g., movement in absence of linguistic stimuli, or following unrelated stimuli) in this design does not allow us to disentangle whether the results in Experiment 2 are the product of an homogenous motor activation for all perspectives or the absence of it. In a more nuanced view, future studies should also clarify the relationship between kinematics and behavioral measures and the magnitude of the same effect at the neurophysiological level. Kinematics results of Experiment 2 might be the product of neural effects similar to Experiment 1 but weaker, which translate into no effect on overt movement execution. Future studies directly comparing the same manipulation with different techniques are highly recommended.

Overall, our results indicate that the influence of language on the motor system is likely not automatic but highly flexible and context dependent. Our results namely showed that the motor system activation was strongly influenced by the used pronoun: we found evidence for it with the YOU pronoun and not with the third pronoun, and we found that the YOU pronoun differently influenced the motor response depending on the verb with which it was combined. The effect was also modulated by the spoken language, since the interaction Verb–Pronoun was not present in German but only in Italian.

The way the motor planning was affected by language in Experiment 1 was undoubtedly interesting, since both perspectives induced by pronouns and chain organization of verbs seemed to be involved. We will first handle the role of perspective and of action verbs in Experiment 1, and then we will discuss why the same effects were not found in Experiment 2.

### Pronouns and Perspective Taking

Results of Experiment 1 clearly reveal that the perspective induced by the pronoun affects the motor system. Specifically, our data show a strong effect of the YOU perspective in modulating both action and interaction verbs, and notably this pattern is present in all our subjects. This complements and extends the results obtained by Brunyé et al. (2009), since we used a motor task and demonstrated that perspective modulates the very first stages of actions planning and subsequently execution. Our preliminary results are also consistent with the previous studies where the strongest compatibility/facilitation effects (Glenberg and Kaschak, 2002; Gianelli et al., 2011) are obtained with sentences using YOU or with the infinitive form of the verbs, where the perspective activated is necessarily the one of the agent.

Our results suggest instead that the perspective elicited while reading the pronoun HE is more abstract and external, so that the motor effects of language processing disappear. This might appear in contrast with the results obtained in a recent behavioral and TMS study by Gianelli and Dalla Volta (2015) who showed a motor facilitation with the use of a passive listening task for third-person Italian sentences. However, in this study the authors used only a passive listening task and implicit agent’s attribution (i.e., no pronoun) and stimulated the motor cortex before the agent’s information was made explicit. In addition, only third-person sentences were presented, with no perspective manipulation. Further studies are thus needed to investigate under which conditions the third person perspective activates an agent perspective and at which degree. Interestingly, what happens for HE seems to be true – at least partially – for the I perspective as well. The I perspective may involve the subject a bit more than the HE perspective. However, overall our results point to the idea that the role of agent is taken when the YOU pronoun is used. In this condition it appears that the participants are called directly into action and then they re-activate the motor pattern of an action from the point of view of the agent. I and HE constitute external and “observational” perspectives but at different degrees. In an inter-subjective framework, as for example in a conversation, the use of the pronoun I normally refers to the presence of a speaker who is reporting the action from his/her point of view, whereas we (i.e., the readers) are recruited as recipients of his/her speech. In the case of the pronoun HE, a radically external perspective is assumed. Consider for instance a situation in which we and another person are talking of the actions of a third person: his/her perspective does not involve us directly.

Overall, the results of Experiment 1 suggest that while comprehending language we activate an inter-subjective framework, as the role of the YOU pronoun with interactive verbs indicate. This happens even if we are not directly involved in communication but simply read linguistic stimuli. The adoption of this frame of reference has a very precocious effect as it differently impacts the early stages of movements planning and execution. The activation of a conversational framework has an interesting theoretical implication. Even if our study showed that under certain conditions action organization (e.g., the motor chains) might be reflected in language (e.g., in Italian), language imposes its own constraints on the way actions are conceived, giving relevance to the YOU perspective in taking the agent’s perspective. In line with evidence on neural re-use of previously built neural structures (Gallese, 2008; Anderson, 2010, 2014), our work shows that language builds on previously formed structures, such as the action chained organization, but also that it strongly constrains and modifies it (Borghi, 2012, for discussion of this issue), as the importance assumed by the YOU perspective clearly demonstrates.

### Verbs and Motor Chains

The motor pattern activated by YOU both with AVs and IVs fits well also with our hypothesis about the organization of actions in motor chains, supporting the notion that an agent’s perspective is activated. In fact, IVs result in a shorter time to velocity peak, so that conversely the deceleration phase is longer. This is coherent with evidence on motor planning and control of a sequence of motor acts (Gentilucci et al., 1997): an increasing accuracy in interaction with the object influences arm velocity profiles by decreasing the velocity peak and lengthening the deceleration phase. In this sense the current motor act is influenced by the requests of the successive act. AVs do not imply any particular request of accuracy since there is not a second motor act to plan: namely, the action ends with the grasping of the object. This is not the case for IVs where more accuracy is requested in order to interact with the object: indeed, the object should be grasped and given to somebody else. One could speculate that participants are particularly accurate also due to the fact that IVs do not simply involve a further motor act compared to AVs, but that they also involve a social dimension, guaranteed by the virtual presence of a recipient. However, our data do not allow us to definitively solve this issue since no direct social manipulation was designed.

### Cross-Linguistic Differences

Once verified that the Italian pronouns influence perspective taking with action verbs, we performed a conceptual replication of the same study in German (Experiment 2), comparable for task and design – e.g., both studies directly manipulate and compare different perspectives in combination with specific verb categories. The reasons why we were interested in performing the same study in another language, and specifically in German, are many. First, we think it is important to conduct cross-cultural studies. In many cases researchers implicitly assume that the phenomena they find hold across different populations, while often this is not the case (for a recent review, see Henrich et al., 2010). To make general claims it is therefore important to investigate whether the same phenomenon holds in different populations. Second, we believe it is crucial not only to realize cross-cultural, but also cross-linguistic studies. The last years have been characterized by a resurgence of interest for linguistic relativity, the idea that natural languages shape the way we think and conceptualize the world (Whorf, 1956; Casasanto, 2008; Reines and Prinz, 2009). Once identified a phenomenon – in our case the fact that the perspective induced by pronouns influences the motor system – it is important to verify to what extent such phenomenon is generalizable across different natural languages. Our results suggest that the interaction Verb–Pronoun we found is not generalizable to German, and this has theoretical implications since our results are in line with the idea that the language we speak can differently influence perspective taking. A third specific reason is related to the specific differences of Italian and German in the use of pronouns, which are mandatory in German but not in Italian. As anticipated in the introduction to Experiment 2, we intended to investigate whether the effects found in Italian was replicated in German, a language where pronouns play a different role. Experiment 2 did not yield the same results and instead pointed to the absence of difference between conditions, in particular pertaining the crucial interaction of verb type and pronoun. We will now discuss the possible reasons underlying such a discrepancy.

The first and more crucial difference between Italian and German and the reason why we performed the second experiment in German pertains the role of the pronouns. While the use of pronouns is mandatory in German, it is not in Italian. Our results showed that the difference in processing action and interaction verbs with the YOU pronoun was present only in Italian. We interpret this difference as due to the fact that, since the use of pronouns in Italian is not necessary, their presence might be perceived as more salient. This suggests that not language *per se*, but different natural languages have a different impact on perspective taking.

One further possible explanation of the difference we found between the two experiments concerns the tense of verbs: the two experiments do not fully overlap, since we used past tense in Italian, and present tense in German. Although the choice of the two tenses was due to pure methodological reasons (e.g., in keeping with the methodology used in Experiment 1 and the relative kinematics analysis) and this factor was not manipulated, literature suggests that different tenses might indeed lead to different motor activations, supporting a flexible view of embodied language processing (e.g., Bergen and Wheeler, 2010; Candidi et al., 2010). From this point of view, it is possible that different verb tenses activate motor resonance at a different degree, making a stronger motor resonance more capable to affect motor behaviors than weaker ones, especially when combined with certain perspectives (e.g., more internal ones). However, we tend to exclude that the effect is due to the different tenses used since we found a stronger modulation of the motor system in Italian, i.e., when we used the past tense, than in German, when we used the present tense.

We tend rather to believe that the most plausible explanation of the differences in results is due to the structural differences between Italian and German language and in particular to how pronouns differently influence perspective taking.

On the other hand, it is worth considering for future research that linguistic differences between the two experiments are not limited to the differences in linguistic stimuli *per se*. Indeed, we did compare not only two sets of stimuli but also two groups of native speakers whose linguistic habits are very different. The degree to which these linguistic habits could have affected their motor behavior and the way they handled the linguistic task, cannot be solved but only pointed out by the exploratory data we made available. The study of embodied language processing so far has focused on few languages (with a predominance of English, Italian, French, Dutch and more limitedly German) and the direct comparison of different languages in the same study is in most cases absent. This seems indeed surprising as one would clearly expect that different linguistic and motor experiences would affect the encoding of the corresponding linguistic labels, and hence the re-activation of these experiences in terms of motor resonance. Similarly, if while comprehending language we activate an inter-subjective framework (as suggested by Experiment 1), this might occur differently in two languages, being more or less flexible in different groups of native speakers. Our exploratory study points out the need for future studies performing direct cross-linguistic comparisons, and when possible comparing different groups of speakers (e.g., native vs. not native). At the same time, we believe that the use of kinematics and hence of motion analysis, could constitute a powerful tool for such comparisons, making it possible to use the same motor tasks regardless of the tested language.

## Ethics Statement

The study did not involve any risk for participants health or wellbeing. Experiment 1 was performed at the University of Bologna, Department of Psychology and was approved by local ethics committee. Experiment 2 was performed at the University of Potsdam, Division of Cognitive Sciences following the local ethics procedures (written informed consent), but being a behavioral study it was exempt by the requirement of formal approval. All participants were informed as to the procedures involved in the experiment and gave their written informed consent.

## Author Contributions

CG and AB designed the experiment, CG and MM collected and analyzed the data, CG and AB wrote the paper.

## Conflict of Interest Statement

The authors declare that the research was conducted in the absence of any commercial or financial relationships that could be construed as a potential conflict of interest.

## Footnotes

1 Following a reviewers’ suggestion, we tested the same parameter with a different normalization procedure (for possible issues connected to the use of normalized parameters, see Whitwell and Goodale, 2013). Namely, instead of normalizing the latency of velocity peak with respect to the reach time (as described in the Section Methods), we used the overall movement time as reference measure. Analyses on this new parameter confirmed the statistical significance of the interaction between Verb and Pronoun [*F*(2,22) = 4.087, *p*= 0.031, η_p_^2^ = 0.3]. Three paired sample *t*-tests (with a corrected *p*-value of 0.02) confirmed the significant difference between AVs and IVs when preceded by the pronoun YOU [*t*(11) = 2.5, *p*= 0.01, with a 4.3% difference between conditions]. On the contrary, no significant effect was detected for conditions involving the pronouns I and HE (*p* = 0.2 and 0.6, respectively)
